# Practice assistants in primary care in Germany – associations with organizational attributes on job satisfaction

**DOI:** 10.1186/1471-2296-14-110

**Published:** 2013-08-05

**Authors:** Amina Gavartina, Stavria Zaroti, Joachim Szecsenyi, Antje Miksch, Dominik Ose, Stephen M Campbell, Katja Goetz

**Affiliations:** 1 Department of General Practice and Health Services Research, University of Heidelberg, Heidelberg, Germany; 2 Centre for Primary Care, Institute of Population Health, University of Manchester, Manchester, UK

**Keywords:** General practice, Practice assistant, Organizational attributes, Job satisfaction, Primary health care

## Abstract

**Background:**

Job satisfaction and organizational attributes in primary care teams are important issues as they affect clinical outcomes and the quality of health care provided. As practice assistants are an integral part of these teams it is important to gain insight into their views on job satisfaction and organizational attributes. The aim of this study was to evaluate the job satisfaction of practice assistants and the organizational attributes within their general practices in Germany and to explore the existence of possible associations.

**Methods:**

This observational study was based on a job satisfaction survey and measurement of organizational attributes in general practices in the German federal state of Baden-Wuerttemberg. Job satisfaction was measured with the 10-item ‘Warr-Cook-Wall job satisfaction scale’. Organizational attributes were evaluated with the 21-items ‘survey of organizational attributes for primary care’ (SOAPC). Linear regression analyses were performed in which each of SOAPC scales and the overall score of SOAPC was treated as outcome variables.

**Results:**

586 practice assistants out of 794 respondents (73.8%) from 234 general practices completed the questionnaire. Practice assistants were mostly satisfied with their colleagues and least of all satisfied with their income and recognition for their work. The regression analysis showed that ‘freedom of working method’ and ‘recognition of work’, the employment status of practice assistants and the mode of practice were almost always significantly associated with each subscale and overall score of SOAPC.

**Conclusions:**

Job satisfaction is highly associated with different aspects of organizational attributes for primary care (‘communication’, ‘decision-making’ and ‘stress’). Consequently, improved job satisfaction could lead to a better-organized primary care team. This implication should be investigated directly in further intervention studies with a special focus on improving the recognition for work and income.

## Background

Within the last years more attention is given on the changing role of practice assistants in primary care. The substitution and diversification of their roles are related to outcomes of patient health, process of care or direct and indirect costs [1]. Moreover, it was found that task substitution to practice assistants supports general practitioners to provide a good care [1-3]. Practice assistants are crucial for providing a good quality of care and positive health outcomes for patients, while also improving the level of service capacity and accessibility [4]. Furthermore, the strengthening of the role of practice assistants in primary care is expected to improve the quality of service provided by physicians via a reduction of their workload enabled by a transfer of responsibilities to practice assistants [5]. Moreover, the changing role could have an impact on perception of own job satisfaction. Job satisfaction is an important issue among health care professionals and is associated with suboptimal health care delivery and poor clinical outcomes, for instance adverse events and reduced patient adherence [6]. Additionally, it is imperative to maintain a high quality of care due to the demographic change, the increase of chronic diseases and continuous medical progress. The demographic change, and with it the increase of chronic diseases, is relevant not only because of the rising health costs but also in terms of personnel resources [7]. These developments, the changing role of practice assistants and their impact on job satisfaction and the demographic change, play an important role in light of prospective personnel retention.

Workforce shortages within the health care system are becoming a generic problem in several countries [8]. The evaluation of job satisfaction plays a significant role in this trend and dissatisfaction leading to a high turnover and a shortage of non-physician employees [9]. This in turn has detrimental effects, with a high turnover leading to a loss of continuity of care [7]. Moreover, it was found that continuity of care could also be influenced by organizational attributes in primary care [10]. However, there is a lack of evidence regarding the relationship of organizational attributes and job satisfaction by practice assistants in primary care. Currently, there are around 398,000 practice assistants in primary care in Germany [11]. They are trained in supportive medical tasks, practice organisation and administration during a 3-year part-time curriculum at a vocational school [12]. Their range of tasks varies from administrative tasks, assisting general practitioners and performing various medical procedures.

The aim of the study was to evaluate the job satisfaction and organizational attributes of practice assistants in general practices in Germany and to explore associations between organizational attributes and job satisfaction.

## Methods

This observational study was based on a job satisfaction survey and measurement of organizational attributes in general practices in the German federal state of Baden- Wuerttemberg.

### Participants

From 2000 randomly selected general practices 234 took part in the job satisfaction survey of practice assistants. From these 234 general practices 794 practice assistants were interested to take part in this study. Practice assistants were asked to fill in a depersonalized paper-based questionnaire and to send it back to the study centre. We provided a free-post envelope, but no further financial incentives for practice assistants.

### Measures

Data collection took place between June 2011 and August 2011. The questionnaire contains the German validated version of the ‘Warr-Cook-Wall (WCW) job satisfaction scale’, developed by Warr et al. and the German validated version of the ‘survey of organizational attributes for primary care’ (SOAPC) [13-15].

The job satisfaction scale consists of ten items rated on a 7-point Likert scale (1 = extreme dissatisfaction to 7 = extreme satisfaction). The questionnaire comprises items concerning the satisfaction with physical working conditions, freedom to choose work methods, colleagues and fellow workers, recognition for work, amount of responsibility, income, opportunity to use one’s abilities, hours of work and amount of variety in the job as well as the overall job satisfaction. A higher overall mean score indicates a higher job satisfaction. The ‘WCW job satisfaction scale’ is also regularly used in a primary care focus as shown in a literature review conducted by van Ham et al. and by other studies [16-18], and can be regarded as a reliable and validated instrument to measure job satisfaction.

The German validated version of SOAPC is composed of 21 items, which are subsumed in four subscales: ‘communication’ consisting of four items, ‘decision-making’ consisting of eight items, ‘stress’ consisting of six items and ‘change’ consisting of three items [14]. Each of the items is rated on a 5-point Likert scale (1 = fully disagree to 5 = fully agree). This version was used in a primary care focus before [15].

Furthermore, socio-demographic data such as age, gender, employment status and practice characteristics were collected. Practice characteristics contained the mode of practice (single-handed and group practices) and the location of the practice (urban, semi-urban and rural located practices).

### Data analysis

The analysis was performed with SPSS version 18.0 (SPSS Inc., Chicago IL, USA). First, a descriptive analysis was undertaken concerning characteristics of the study population. Furthermore, a descriptive analysis of job satisfaction scale and scales of organizational attributes were conducted. The means, standard deviations and 95% confidence intervals of these aspects are reported. Finally, multivariate linear regression analyses for each of the organizational attributes were carried out. Each of the four scales of SOAPC and the overall score of SOAPC were treated as outcome variables, the individual and practice characteristics and the different items of the job satisfaction scale were used as independent variables. An alpha level of p < 0.05 was used for tests of statistical significance.

### Ethical approval

The study was fully approved by the ethics committees of the Medical Faculty of the University of Heidelberg (S137/2011).

## Results

586 practice assistants out of 794 respondents (73.8%) completed the questionnaire. Almost all practice assistants were female (99.5%) with a mean age of 39.1 years (SD = 12.0). 44% of the practice assistants worked full time. Over 40% of the general practices were single handed and 30.6% were located in rural areas.

### Evaluation of job satisfaction and organizational attributes

Practice assistants were mostly satisfied with ‘colleagues and fellow workers’ (mean = 5.87) and least satisfied with the ‘recognition of their work’ (mean = 5.07) and their ‘income’ (mean = 3.89). The mean for the ‘overall job satisfaction’ was 5.74 (SD = 1.19) meaning that practice assistants are satisfied with their job situation in general. The overall mean score for the factors of organizational attributes for practice assistants was 3.70 (SD = 0.48) indicating that there exist a functioning organizational culture within practices. However the subscale ‘history of change’ with a rating of 3.12 (SD = 0.79) as the lowest mean showed that practice assistants thought that there were not sufficient changes in the work organization and teamwork. Nevertheless ‘decision making’ was rated the highest with a mean of 3.95 (SD = 0.64) showing that practice assistants thought that the practice works as a team concerning decision-making and the development of improvements regarding the quality of care within the practices. The subscale ‘stress’ had a mean of 3.53 (SD = 0.73). A lower mean in this subscale means less stress. Practice assistants were moderately stressed meaning that the given working conditions are only moderately exhausting. Details are given in Table 1.

**Table 1:** Descriptive statistics factors of job satisfaction and of organizational attributes of practice assistants

	**Mean (SD)**	**CI (95%)**
**Factors of job satisfaction**^**1**^		
Physical working condition	5.18 (1.32)	5.06 – 5.30
Freedom of working method	5.20 (1.35)	5.08 – 5.33
Colleagues and fellow workers	5.87 (1.28)	5.76 – 5.99
Recognition for work	5.07 (1.47)	4.94 – 5.21
Amount of responsibility	5.38 (1.33)	5.25 – 5.50
Income	3.89 (1.79)	3.73 – 4.06
Opportunity to use abilities	5.26 (1.26)	5.14 – 5.37
Hours of work	5.34 (1.49)	5.20 – 5.48
Amount of variety in job	5.49 (1.22)	5.38 – 5.60
Overall job satisfaction	5.74 (1.19)	5.63 – 5.85
**Factors of organizational attributes**^**2**^		
Communication	3.92 (0.48)	3.86 – 3.98
Decision making	3.95 (0.64)	3.89 – 4.00
Stress	3.53 (0.73)	3.46 – 3.59
History of change	3.13 (0.79)	3.06 – 3.20
Overall score	3.70 (0.48)	3.66 – 3.75

*SD* Standard deviation, *CI* Confidence interval.

^1^range from 1 “extreme dissatisfaction” to 7 “extreme satisfaction”.

^2^range from 1 “fully disagree” to 5 “fully agree”.

### Factors associated with the outcome variables: organizational attributes

The associations between the predictors of individual and practice characteristics and factors job satisfaction concerning the organizational attributes are presented in Table 2.

**Table 2:** Associations on outcome variables of organizational attributes in primary care practices (results of linear regression analyses, under specification of standardized beta coefficient and 95% confidence interval (CI), α = 5%)

	**Factors of organizational attributes of primary care practices**				
	**Communication**	**Decision-making**	**Stress**	**History of change**	**Overall score**
	β (p-value*)	β (p-value*)	β (p-value*)	β (p-value*)	β (p-value*)
**Practice characteristics**					
Mode of practice (0 = single; 1 = group)	**−0.107 (0.01)**	−0.016 (0.66)	**−0.154 (<0.01)**	**0.160 (0.01)**	−0.068 (0.07)
Location of practice (1 = urban; 2 = semi urban; 3 = rural)	−0.024 (0.52)	−0.015 (0.69)	0.055 (0.17)	0.024 (0.62)	0.018 (0.63)
**Individual characteristics**					
Age	−0.011 (0.80)	0.058 (0.17)	**−0.155 (0.01)**	0.096 (0.07)	−0.021 (0.62)
Employment status (0 = full-time worker; 1 = part-time worker)	**0.090 (0.04)**	0.080 (0.06)	**0.138 (0.01)**	−0.056 (0.30)	**0.110 (0.01)**
**Different factors of job satisfaction**					
Physical working condition	−0.033 (0.53)	−0.015 (0.77)	**0.356 (<0.01)**	−0.085 (0.19)	**0.121 (0.02)**
Freedom of working method	**0.127 (0.02)**	**0.243 (<0.01)**	0.034 (0.54)	0.123 (0.06)	**0.202 (<0.01)**
Colleagues and fellow workers	**0.397 (<0.01)**	0.082 (0.09)	0.038 (0.46)	−0.106 (0.08)	**0.141 (0.01)**
Recognition for work	**0.211 (<0.01)**	**0.283 (<0.01)**	0.081 (0.17)	0.006 (0.93)	**0.238 (<0.01)**
Amount of responsibility	−0.085 (0.15)	**−0.124 (0.03)**	−0.067 (0.28)	0.006 (0.94)	**−0.119 (0.04)**
Income	0.063 (0.16)	−0.007 (0.88)	0.043 (0.37)	0.048 (0.40)	0.043 (0.33)
Opportunity to use abilities	0.058 (0.36)	**0.204 (0.01)**	0.070 (0.31)	−0.096 (0.23)	**0.127 (0.04)**
Hours of work	−0.044 (0.38)	−0.020 (0.69)	0.013 (0.80)	−0.027 (0.67)	−0.021 (0.66)
Amount of variety in job	0.026 (0.65)	0.046 (0.40)	−0.092 (0.12)	0.095 (0.18)	0.015 (0.79)
Overall job satisfaction	−0.007 (0.91)	0.046 (0.50)	0.095 (0.20)	−0.057 (0.51)	0.050 (0.46)
**Pseudo R**^**2**^	**0.389**	**0.405**	**0.298**	**0.042**	**0.424**

*Statistical significance of difference: P < 0.05.

Three of the five regression models explained more than 35% (R^2^ > 0.35) of the variance of the outcome variables. These three subscales were ‘communication’, ‘decision-making’, and the ‘overall score’. As an example, the predictors that were significantly associated with the outcome ‘communication’ were three factors of job satisfaction: ‘freedom of working method’ (β = 0.127), ‘colleagues and fellow workers’ (β = 0.397) and ‘recognition for work’ (β = 0.211). The ‘overall job satisfaction’ was no important predictor for each subscale and overall score of SOAPC. Of the 14 independent variables that were entered into the regression models, two items of the job satisfaction scale being ‘freedom of working method’ and ‘recognition of work’, the employment status of practice assistants and the mode of practice were almost always significantly associated with each subscale and overall score of SOAPC.

## Discussion

The results of the study show how organizational attributes and socio-demographic characteristics are associated with the job satisfaction of practice assistants in German primary care settings. As there is limited published research in this field so far this study contributes important issues to a field with increasing importance.

Higher scores of the different aspects of job satisfaction compared to our data were found in studies which have also evaluated job satisfaction of practice assistants in general practices with the Warr-Cook-Wall questionnaire [17,18]. A possible explanation for higher scores could be the different study populations. All of the respondents from these previous studies were members of general practices participating in a quality management program. In a study conducted by Vail et al. it was also shown that, despite a high overall satisfaction, practice assistants were dissatisfied with their income, status and career progression [19]. Practice assistants in Australia indeed showed higher scores for overall job satisfaction than in our study, but were also less satisfied with their ‘income’ and ‘recognition for their work’ [20].

One solution to the noticeable lack of recognition could be the reallocation of tasks giving practice assistants more responsibilities, which might not only strengthen the importance of their job but also reduce the workload of general practitioners. It was shown that task substitution could contribute to an improvement of quality of care as well as patient outcomes such as patient adherence to treatment, patient quality of life and patient satisfaction [21,22]. Moreover, a study of Dini et al. showed that 48% of general practitioners in Germany inclined to delegate home visit tasks to their practice assistants. The main disadvantage here was the related costs for training of the practice assistants, while the ability to save time was named as the main advantage [23]. To realise task substitution in Germany there are for example the so called ‘AGnES-model projects’ in different federal states, which transfer medical duties and house visits to qualified practice assistants. Results of these model projects showed that general practitioners feel exonerated and patient compliance is improved through AGnES-employees [24].

As our data suggests colleagues make an important contribution to job satisfaction. These results are in accordance with other studies. Zangaro et al. for example showed that through a high satisfaction with colleagues stress could be reduced leading to a more positive work environment [25]. Moreover, a review of Van Laar et al. emphasized that in most of the researched 17 countries medical staff was stressed and incriminated. It was assumed that the nature of work, numerous changes in organizational fields and the growing workload lead to worse physical and mental conditions which could deteriorate the quality of patient care [26]. Moreover, job dissatisfaction implicated more stress and burnout amongst health care workers [27]. This shows the importance of research evidence concerning organizational culture, job satisfaction and working conditions of practice assistants. Our study found that the level of ‘stress’ was moderate, with a lower mean indicating a lower stress level. Moreover, there was a significant association between this subscale and the age of the participants. A lower level of stress within the practices is associated with a younger age of the practice assistants, group practices and practices with a higher level of satisfaction with physical working condition. This emphasizes the importance of the general trend towards more group practices as a contribution to a reduction of the level of stress [28].

In our study we found a strong and significant association between organizational attributes, job satisfaction and socio-demographic characteristics throughout the regression models. This means that job satisfaction affects changes in the organizational culture within a general practice concerning, for example, decision making, improving working conditions, teamwork, provided quality of care, reducing stress and exhaustion. In further intervention studies should be investigated to establish whether or not interventions could have an impact on both organizational culture and job satisfaction. Additionally, improved job satisfaction could lead to a better organized primary care team. This association, which was also shown in other studies [15,29], is important as team-level job satisfaction is a telling predictor for the effectiveness of how primary care teams manage patient care [15]. Kuokkanen et al. found that organizational commitment and job satisfaction are essential factors for empowerment. With empowerment they mean “the process of personal growth and development”, which is important in the context of the attractiveness of the profession as well as the development of care, education and management [30]. Other factors instrumental for empowerment include a low-hierarchy organization, a teamwork oriented approach within a practice, sufficient capabilities and a management which creates opportunities [31]. An important contribution to the job satisfaction of nurses could be set in a meta-analysis from 1993 performed by Blegen et al., which revealed that nurses’ job satisfaction was strongly associated with declined work stress, organizational commitment, communication and recognition for work [32]. These findings are exposed and confirmed for practice assistants by our study. It should be noted that ‘history of change’ had only an explained variance of 4.2%. The reason for this might be due to the limited options of practice assistants to initiate changes as the general practitioner makes most of the final decisions. To confirm this assumption and to address the question regarding the distribution of decision-making in a team, further studies need to be conducted.

### Strengths and limitations

A main strength of our study is that we used internationally validated measures for the evaluation of organizational attributes and job satisfaction of practice assistants [13,14]. Furthermore, there is little research for organizational attributes of practice assistants, particularly for Germany. Therefore, our study provides an important addition to this field of research. However, without being able to collect data from a broader range of socio-demographic characteristics of practice assistants only limited conclusions regarding this area are possible. In addition, as this was an exploratory study; p values should be interpreted carefully. Significant results might be due to chance and will need to be confirmed in further targeted studies.

## Conclusions

Different items of job satisfaction, such as ‘freedom of working method’, ‘colleagues and fellow workers’ and ‘recognition for work’, affect specific organizational attributes such as ‘communication’, ‘decision-making’ and ‘stress’. Furthermore, job satisfaction affects differences in the organizational culture concerning of decisions made by the whole team, working conditions, teamwork, the provided quality of care and the reduction of stress, chaos and exhaustion. Therefore, an improved job satisfaction could lead to a better-organized primary care team. Based on this finding the existing connection between organizational attributes with different items of job satisfaction should be investigated in further intervention studies. This could provide an important basis for developing more and wider interventions concerning the establishment and improvement of satisfied, extended and multidisciplinary primary care teams. Practice assistants are an essential part of primary care teams and therefore, it is important to evaluate their job satisfaction. However, the recognition for their work and their income should be improved. The reassigning of their tasks from administrative functions to more health care based tasks linked with more responsibility and an increase of job satisfaction which should be investigated in further studies.

## Competing interests

The authors declare that they have no competing interests.

## Authors’ contributions

KG, AM and JS initiated and designed the study. KG coordinated the study. AG, SZ and KG carried out data analysis. AG and KG wrote the manuscript. All authors (AG, SZ, JS, AM, DO, SC, KG) commented on the draft and approved the final version of the manuscript.

## Pre-publication history

The pre-publication history for this paper can be accessed here:

http://www.biomedcentral.com/1471-2296/14/110/prepub
